# Renal Artery Stent-Graft Implantation Using the Retrograde Approach in a Patient Who Developed a Renal Artery Aneurysm after Thoracoabdominal Aortic Repair

**DOI:** 10.70352/scrj.cr.24-0121

**Published:** 2025-04-18

**Authors:** Yutaka Matsubara, Tadashi Furuyama, Toshihiro Onohara

**Affiliations:** Department of Vascular Surgery, NHO Kyushu Medical Center, Fukuoka, Fukuoka, Japan

**Keywords:** renal artery aneurysm, stent graft, retrograde approach, hostile abdomen, Marfan syndrome

## Abstract

**INTRODUCTION:**

Herein, we report a patient who underwent stent-graft implantation using the retrograde approach for a renal artery aneurysm.

**CASE PRESENTATION:**

The patient was a 48-year-old man who underwent total arch replacement, thoracoabdominal aortic repair, aortic root replacement, and thoracic endovascular aortic repair for Marfan syndrome. A right renal artery aneurysm developed at the prosthetic graft anastomosis during observation. First, stent-graft implantation using the antegrade approach was performed. However, the delivery system could not be advanced to the right renal artery because of prosthetic graft kinking. Therefore, the procedure was discontinued. Next, the retrograde approach was used. A right hypochondral oblique incision was made. The right renal artery was exposed with the retroperitoneal approach, and a stent graft was retrogradely inserted into the renal artery and deployed between the prosthetic graft and the distal right renal artery to cover the aneurysm. The patient was followed up for 3 years after the surgery, and he did not develop any aneurysm.

**CONCLUSIONS:**

Renal artery stent graft implantation using the retrograde approach can be a treatment option for renal artery aneurysms in patients with a hostile abdomen.

## Abbreviation

AAA
: abdominal aortic aneurysm

## INTRODUCTION

Marfan syndrome is associated with the development of multiple aneurysms, and complex surgical repairs are occasionally required.^1,2)^ The collagen tissues of patients with Marfan syndrome are fragile. Thus, another aneurysm can form after surgical repairs.^3,4)^ Due to the high risks of re-do after surgical repairs,^3,5)^ endovascular surgeries are likely selected. These types of surgeries require appropriate vascular access. Herein, we report a patient who underwent multiple aortic surgeries and who did not have an appropriate access. In this case, stent-graft implantation using the retrograde approach was considered for a renal artery aneurysm.

## CASE PRESENTATION

### Patient

**Fig. 1:**
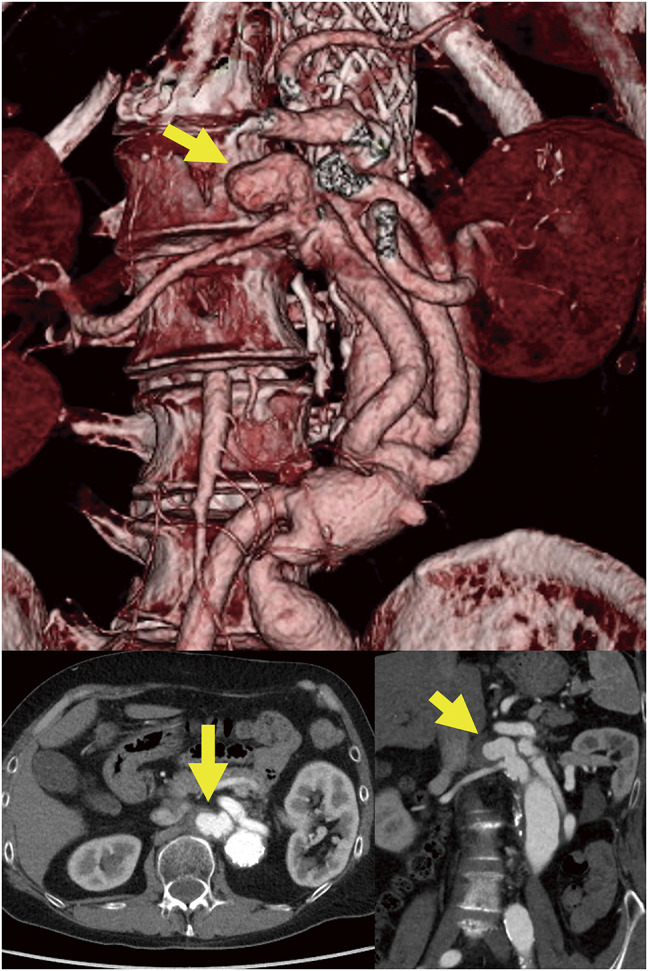
Three-dimensional cardiac computed tomography angiography revealed a right renal artery aneurysm at the prosthetic anastomosis site (arrow). The patient had previously undergone thoracoabdominal aortic repair. The diameter of the right renal artery was 6 mm, and its length was 70 mm. The renal branch of prosthetic graft was 10 mm in diameter and 95 mm in length.

The patient was a 48-year-old man who had undergone total arch replacement, thoracoabdominal aortic repair, aortic root replacement, and thoracic endovascular aortic repair attributed to Marfan syndrome. Warfarin had been administered. In the previous operations, a left intercostal incision and median abdominal incisions were made. The thoracoabdominal aorta was exposed retroperitoneally and repaired. The celiac, superior mesenteric, and bilateral renal arteries were reconstructed. An abdominal aortic aneurysm (AAA) was developed 18 years after thoracoabdominal aortic repair, and the patient underwent AAA repair via median laparotomy. During follow-up, computed tomography scans revealed the development of a right renal artery aneurysm at the prosthetic graft anastomosis (**Fig. 1**). Because the patient had undergone multiple laparotomies for thoracoabdominal and abdominal aortic repairs, stent-graft implantation was selected due to the hostile abdomen.

### Procedures

**Fig. 2:**
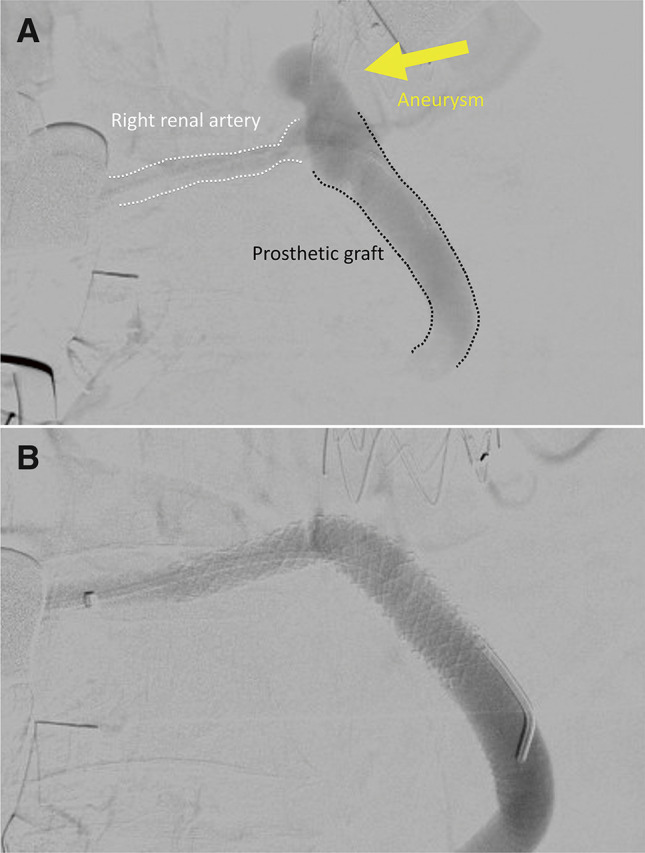
(**A**) Angiography showing a right renal artery aneurysm at the prosthetic anastomosis site (arrow). (**B**) Final angiography revealing the disappearance of the aneurysm after stent-graft implantation. The patient was in supine position, and the C-arm angle was 0°.

**Fig. 3:**
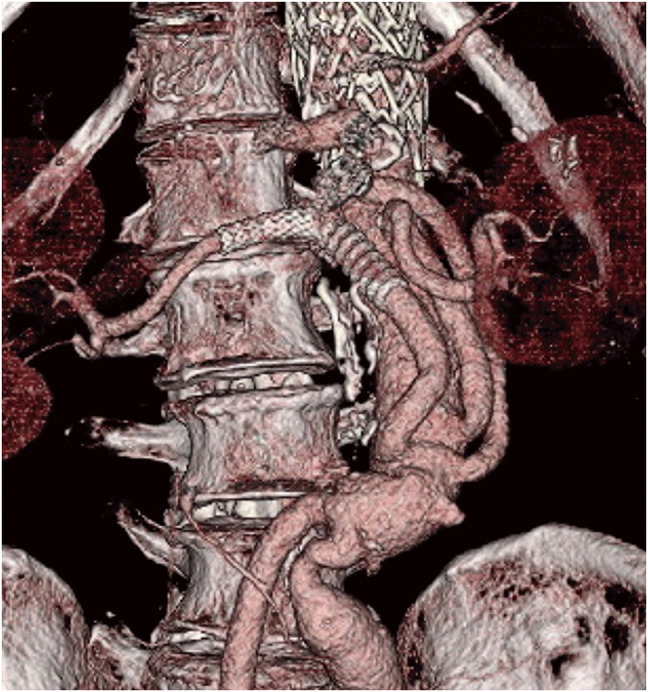
Three-dimensional cardiac computed tomography angiography showing the disappearance of the aneurysm after stent-graft implantation.

First, stent-graft implantation using the antegrade approach was attempted. However, the delivery system could not be advanced to the right renal artery because the origin of the right renal prosthetic branch was kinked. Therefore, the procedure was abandoned. Next, the retrograde approach was used. A right hypochondral oblique incision was made. The right renal artery was exposed using the retroperitoneal approach after Kocherization. A 7-Fr, 55-cm sheath was retrogradely inserted into the distal renal artery and advanced to the anastomosed prosthetic graft over a 0.035-in. stiff guide wire. Angiography revealed a right renal artery aneurysm (**Fig. 2A**). The 7 × 79-mm Viabahn VBX (W.L. Gore, Newark, DE, USA) was deployed between the prosthetic graft and the distal right renal artery to cover the aneurysm. Final angiography showed no evidence of endoleakage in the aneurysm (**Fig. 2B**). During follow-up, the patient underwent computed tomography scan, and he did not develop aneurysms 3 years after the surgery (**Fig. 3**).

## DISCUSSION

Stent-graft implantation using the retrograde approach was performed for a renal artery aneurysm. In this case, the antegrade approach was challenging to implement due to the kinked prosthetic graft anastomosed to the renal artery. In such cases, the retrograde approach can be an option for renal artery stent implantation. Even after thoracoabdominal aortic repair, the retrograde renal artery approach was safely utilized.

Stent-graft implantation is a good procedure, and the retroperitoneal approach is recommended for aneurysm repair in patients with a hostile abdomen.^6,7)^ A hostile abdomen can have severe adhesions, making it difficult to expose the arteries. In this case, the patient had undergone multiple surgical repairs for thoracic aorta aneurysm, thoracoabdominal aortic aneurysm, and AAA. Consequently, surgical repair of the renal artery aneurysm repair was challenging. The renal artery aneurysm could not be exposed for surgical repair. Nevertheless, the combined retroperitoneal and retrograde approach was effective in this case.

Although the long-term outcomes of renal stent-graft implantation are not clearly elucidated, the indications for the use of stent grafts for visceral aneurysms are expanding.^8)^ The guidelines recommend surgical repair for renal artery aneurysms. Nevertheless, endovascular treatments are the primary choice for other visceral aneurysms.^9)^ Stent-graft implantation has anatomical limitations, including appropriate length, diameter, and angulation of landing zones. Stent-graft implantation for renal artery aneurysm is beneficial only for a limited number of patients. Furthermore, it remains a secondary option; however, it can be useful in high-risk surgical patients.

## CONCLUSION

Herein, we present the use of renal artery stent-graft implantation with the retrograde approach in a patient who underwent thoracoabdominal aortic repair. Thus, this procedure can be a treatment option for renal artery aneurysms.

## ACKNOWLEDGMENTS

None.

## DECLARATIONS

### Funding

None.

### Authors’ contributions

Conception and design: YM, TF, TO

Analysis and interpretation: YM, TF, TO

Data collection: YM

Writing the article: YM

Critical revision of the article: TF, TO

All authors have reviewed and approved the final manuscript, and each author agrees to take responsibility for all aspects of the research.

### Availability of data and materials

Not applicable.

### Ethics approval and consent to participate

This work does not require ethical considerations or approval.

### Consent for publication

Informed consent was obtained from the patient.

### Competing interests

The authors declare that they have no competing interests.
